# Supplementary material for: Novel Driver Strength Index highlights important cancer genes in TCGA PanCanAtlas patients
Source: PeerJ. 2022 Aug 11;10:e13860. doi: 10.7717/peerj.13860 (PMC9375969; doi:10.7717/peerj.13860)
Supplement: Supplemental Information 2 [file peerj-10-13860-s002.zip › COHORTS/genes plots/detailed/2021_8_16_14_9_distribution_events_detailed_females_6.pdf]

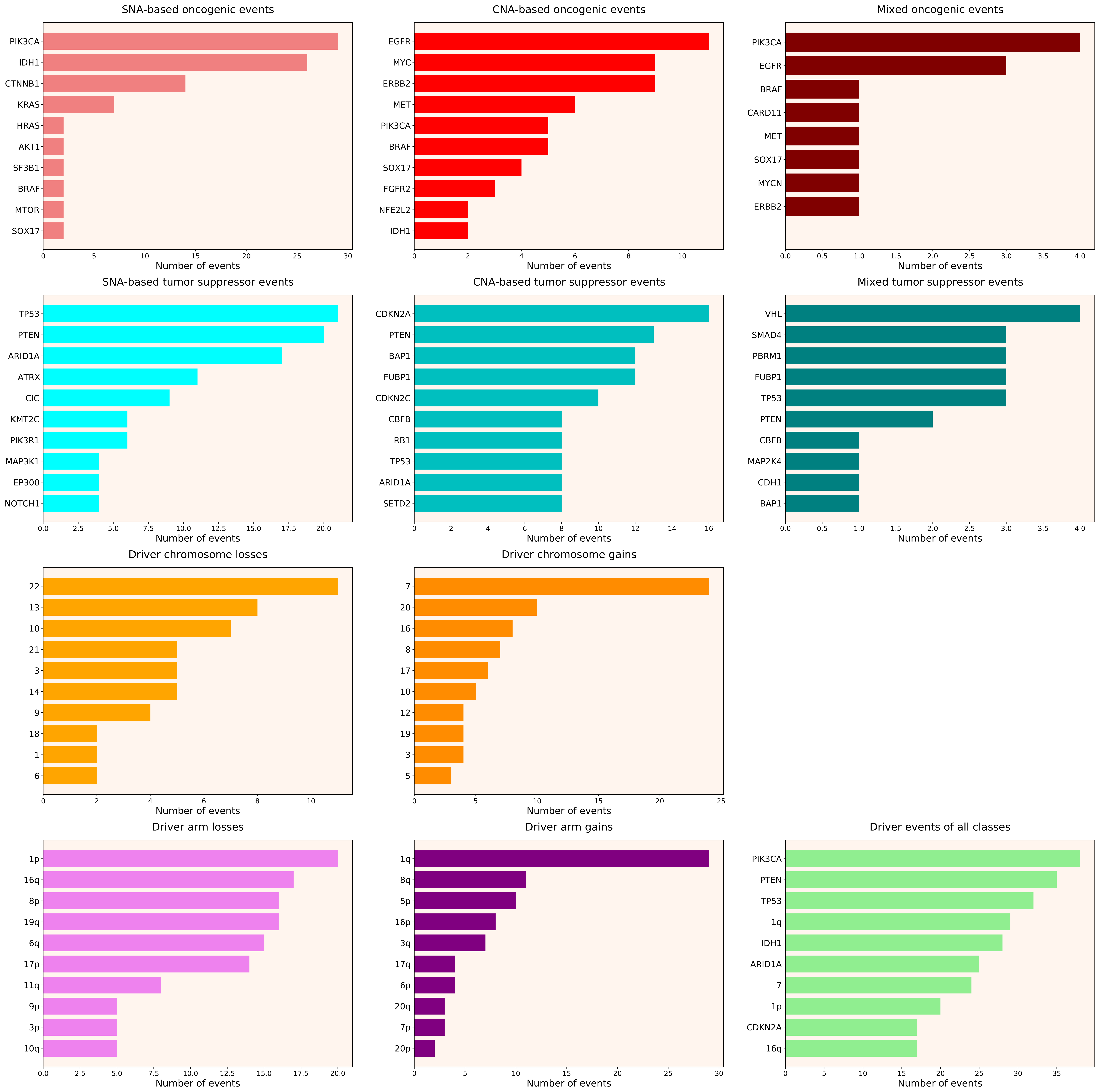

SNA-based oncogenic events
PIK3CA
IDH1
CTNNB1
KRAS
HRAS
AKT1
SF3B1
BRAF
MTOR
SOX17
Number of events
CNA-based oncogenic events
EGFR
MYC
ERBB2
MET
PIK3CA
BRAF
SOX17
FGFR2
NFE2L2
IDH1
Number of events
Mixed oncogenic events
PIK3CA
EGFR
BRAF
CARD11
MET
SOX17
MYCN
ERBB2
Number of events
SNA-based tumor suppressor events
TP53
PTEN
ARID1A
ATRX
CIC
KMT2C
PIK3R1
MAP3K1
EP300
NOTCH1
Number of events
CNA-based tumor suppressor events
CDKN2A
PTEN
BAP1
FUBP1
CDKN2C
CBFB
RB1
TP53
ARID1A
SETD2
Number of events
Mixed tumor suppressor events
VHL
SMAD4
PBRM1
FUBP1
TP53
PTEN
CBFB
MAP2K4
CDH1
BAP1
Number of events
Driver chromosome losses
22
13
10
21
3
14
9
18
1
6
Number of events
Driver chromosome gains
7
20
16
8
17
10
12
19
3
5
Number of events
Driver arm losses
1p
16q
8p
19q
6q
17p
11q
9p
3p
10q
Number of events
Driver arm gains
1q
8q
5p
16p
3q
17q
6p
20q
7p
20p
Number of events
Driver events of all classes
PIK3CA
PTEN
TP53
1q
IDH1
ARID1A
7
1p
CDKN2A
16q
Number of events
